# Cortical grey matter volume differences in children with developmental coordination disorder compared to typically developing children

**DOI:** 10.3389/fnhum.2024.1276057

**Published:** 2024-05-17

**Authors:** Myrah Malik, Alexander Weber, Donna Lang, Tamara Vanderwal, Jill G. Zwicker

**Affiliations:** ^1^Graduate Programs in Rehabilitation Science, University of British Columbia, Vancouver, BC, Canada; ^2^Brain, Behaviour, & Development Theme, BC Children’s Hospital Research Institute, Vancouver, BC, Canada; ^3^Department of Pediatrics, University of British Columbia, Vancouver, BC, Canada; ^4^Department of Radiology, University of British Columbia, Vancouver, BC, Canada; ^5^Department of Psychiatry, University of British Columbia, Vancouver, BC, Canada; ^6^Department of Occupational Science & Occupational Therapy, University of British Columbia, Vancouver, BC, Canada

**Keywords:** developmental coordinator disorder, motor skills disorder, children, MRI, brain structure, voxel-based morphometry, grey matter

## Abstract

**Introduction:**

The cause of Developmental Coordination Disorder (DCD) is unknown, but neuroimaging evidence suggests that DCD may be related to altered brain development. Children with DCD show less structural and functional connectivity compared to typically developing (TD) children, but few studies have examined cortical volume in children with DCD. The purpose of this study was to investigate cortical grey matter volume using voxel-based morphometry (VBM) in children with DCD compared to TD children.

**Methods:**

This cross-sectional study was part of a larger randomized-controlled trial (ClinicalTrials.gov ID: NCT02597751) that involved various MRI scans of children with/without DCD. This paper focuses on the anatomical scans, performing VBM of cortical grey matter volume in 30 children with DCD and 12 TD children. Preprocessing and VBM data analysis were conducted using the Computational Anatomy Tool Box-12 and a study-specific brain template. Differences between DCD and TD groups were assessed using a one-way ANOVA, controlling for total intracranial volume. Regression analyses examined if motor and/or attentional difficulties predicted grey matter volume. We used threshold-free cluster enhancement (5,000 permutations) and set an alpha level of 0.05. Due to the small sample size, we did not correct for multiple comparisons.

**Results:**

Compared to the TD group, children with DCD had significantly greater grey matter in the left superior frontal gyrus. Lower motor scores (meaning greater impairment) were related to greater grey matter volume in left superior frontal gyrus, frontal pole, and right middle frontal gyrus. Greater grey matter volume was also significantly correlated with higher scores on the Conners 3 ADHD Index in the left superior frontal gyrus, superior parietal lobe, and precuneus. These results indicate that greater grey matter volume in these regions is associated with poorer motor and attentional skills.

**Discussion:**

Greater grey matter volume in the left superior frontal gyrus in children with DCD may be a result of delayed or absent healthy cortical thinning, potentially due to altered synaptic pruning as seen in other neurodevelopmental disorders. These findings provide further support for the hypothesis that DCD is related to altered brain development.

## 1 Introduction

Developmental Coordination Disorder (DCD) is defined by motor abilities that are below expectations for the child’s chronological age in the absence of any underlying neurological, visual, or intellectual condition that could better explain the motor difficulties (American Psychiatric Association, 2013). The motor deficit significantly affects activities of daily living, school, work, leisure, and play and can have an adverse impact on mental health and quality of life (Zwicker et al., 2012; Caçola et al., 2016; Zwicker et al., 2017; Izadi-Najafabadi et al., 2019; Karras et al., 2019). The motor difficulties and secondary consequences of DCD often persist into adulthood (Kirby et al., 2014). Children with DCD are more likely than typically developing (TD) children to have attentional difficulties, with over 50% of children with DCD having a co-occurring ADHD diagnosis (Dewey et al., 2002; Fliers et al., 2010).

Neurodevelopmental disorders such as DCD are a heterogenous group of conditions which are thought to be due to impaired growth, development, or function of the central nervous system (CNS) (American Psychiatric Association, 2013). This has led researchers to try to identify brain-based differences in DCD through functional and structural brain imaging studies (Brown-Lum and Zwicker, 2015; Biotteau et al., 2016). Multiple functional studies have identified group-level differences in parietal and frontal regions (Kashiwagi et al., 2009; Zwicker et al., 2010, 2011; McLeod et al., 2014), although these findings have not been consistent. Fewer studies have investigated differences in brain structure. There have been reports of thinner right medial orbitofrontal cortices alongside greater clustering coefficient alterations in the structural connectome of the lateral orbitofrontal cortex in children with DCD (Langevin et al., 2014; Caeyenberghs et al., 2016), but empirical volumetric evidence is sparse in this population. A structural neuroimaging study conducted by Reynolds et al. (2017) found that children with DCD showed significant decrease in grey matter in the frontal lobe of the right hemisphere, and a recent study showed decreased grey matter volume in parts of the cerebellum (Gill et al., 2022). Overall, the number of structural studies in DCD is low, and heterogeneity in sample ages, inclusion criteria, and methodologies used mean there is still much to be learned about structural morphology in children with DCD.

The parietal and frontal lobes have been proposed as one of the correlates of motor impairments in DCD, mainly due to their respective roles in visuospatial information and higher-order cognitive functions (e.g., working memory, organizing/planning). The purpose of this study was to test for potential grey matter volume differences using voxel-based morphometry in children with DCD compared to TD children. We also examined correlations between grey matter volume and clinical measures of motor function and attention difficulties. We hypothesized that children with DCD would have: (1) lower grey matter volume in parietal and frontal regions compared to TD peers; and (2) positive correlations between grey matter volume and motor function and attentional performance.

## 2 Materials and methods

### 2.1 Study design

**Figure 1:**
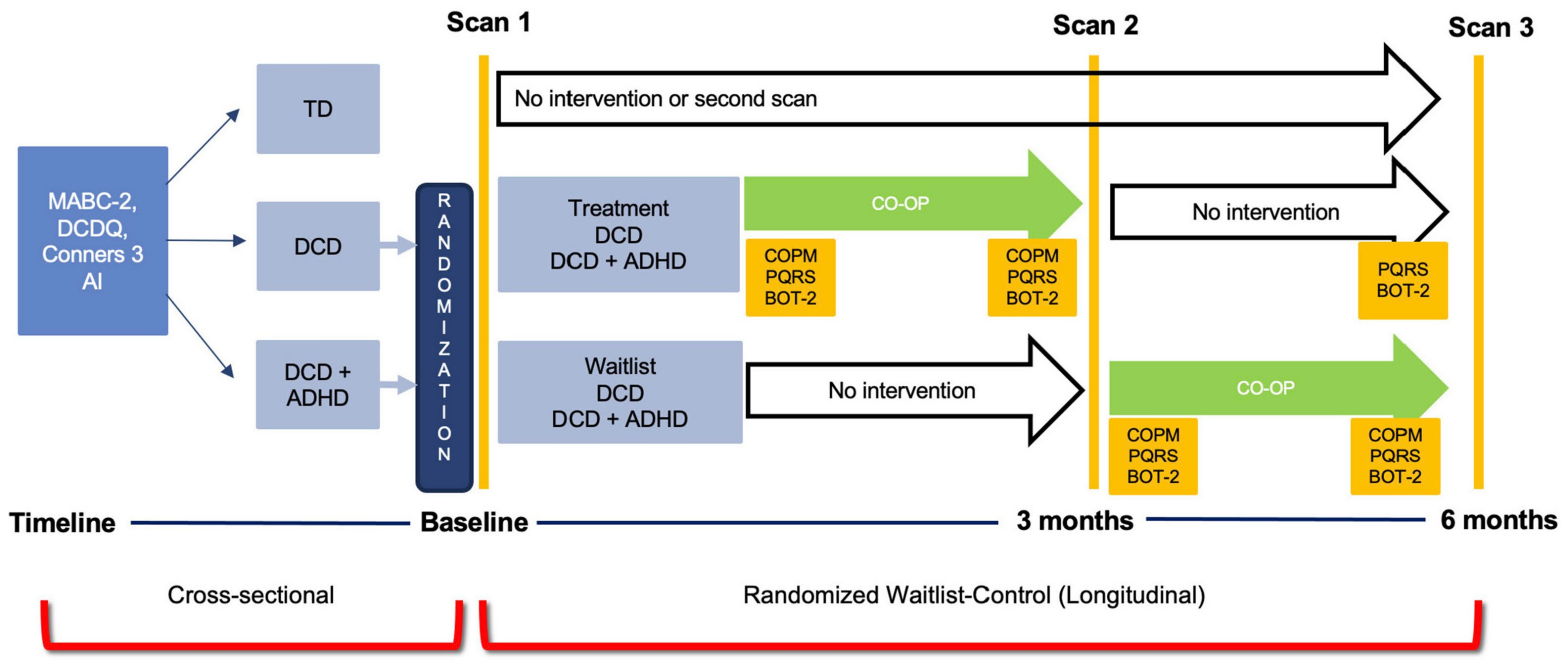
Study Design. ADHD, attention deficit hyperactivity disorder; BOT-2, Bruininks-Oseretsky Test of Motor Proficiency – 2nd ed.; Conners 3 AI, Conners 3 ADHD Index; COPM, Canadian Occupational Performance Measure; CO-OP, Cognitive Orientation to Occupational Performance; DCD, developmental coordination disorder; DCDQ, Developmental Coordination Disorder Questionnaire; MABC-2, Movement Assessment Battery for Children- 2nd ed.; PQRS, Performance Quality Rating Scale; TD, typically developing children.

The current investigation was part of a larger cross-sectional study and randomized waitlist-control trial that used multiple brain imaging modalities (ClinicalTrials.gov ID: NCT02597751). For the purpose of this analysis, data collected as part of the cross-sectional study were used to investigate differences in grey matter volume in three groups of children: DCD, DCD and co-occurring attention deficit hyperactivity disorder (DCD + ADHD), and TD children (Figure 1). Approval was obtained by UBC Children’s and Women’s Research Ethics Board (#H14-00397). After screening and recruitment, parents or legal guardians provided written consent and children assented to participate in the study.

### 2.2 Participants

A convenience sampling method was used to recruit 8-12-years old participants. Children with DCD and DCD + ADHD were recruited from Dr. Zwicker’s research-integrated DCD Clinic at Sunny Hill Health Centre for Children, BC Children’s Hospital ADHD Clinic, caseloads of occupational and/or physical therapists from Sunny Hill and the Vancouver Regional Pediatric Team, and the community. TD children were recruited through advertisements in Vancouver schools and community centres, and by word-of-mouth.

Children were assessed by a registered occupational therapist or trained graduate student to ensure they met the inclusion criteria of the study. Children with DCD were identified according to the Diagnostic Statistical Manual 5th edition (DSM-5) diagnostic criteria (American Psychiatric Association, 2013): (1) a score ≤ 16th percentile on the Movement Assessment Battery for Children – 2nd edition (MABC-2) (Henderson et al., 2007); (2) a score in the suspected or indicative range on the DCD Questionnaire (DCDQ) (Wilson and Crawford, 2007); (3) parent-reported motor difficulties from a young age; and (e.g., cerebral palsy, criteria, intellectual disability) (4) no other medical condition that could explain motor difficulties as per parent-report, clinical observation, and/or medical exam. For the DCD + ADHD group, all the above were met in addition to parent report of an ADHD diagnosis. Given that attention difficulties are common in children with DCD even without ADHD (Dewey et al., 2002), the Conners 3 ADHD Index was used to measure ADHD symptomatology in all participants (Conners, 2009). The control group (TD children) included children 8-12-years old with no history of motor difficulties and a MABC-2 score ≥ 25th percentile. Exclusion criteria included being born preterm (gestational age<37 weeks) or diagnosed with any other neurodevelopmental disorder, such as autism spectrum disorder. Children assigned to the TD group were excluded if they were diagnosed with ADHD. Additionally, children with metal in their bodies (e.g., braces) or with a history of claustrophobia were excluded from participation in the study.

### 2.3 Clinical measurements

The following measures – MABC-2, DCDQ, and Conners 3 ADHD Index – were used to describe the characteristics of the sample for each group.

#### 2.3.1 Movement Assessment Battery for Children - 2nd edition (MABC-2)

The MABC-2 is designed for children (ages 3 to 16 years old) (Henderson et al., 2007) and is the most widely used measure to identify children with DCD (Blank et al., 2019). The MABC-2 assesses a child’s performance in eight motor tasks in three areas of motor performance: (1) manual dexterity; (2) aiming and catching; and (3) balance (Henderson et al., 2007). Raw scores are translated to age-related percentile norms where a lower score indicates greater motor difficulties. The MABC-2 has an internal consistency of α = 0.90, excellent test–retest reliability (ICC = 0.97) and good factorial and construct validity (Schulz et al., 2011; Wagner et al., 2011; Wuang et al., 2012; Psotta and Abdollahipour, 2017). The assessment takes about 30 min to administer and can be administered by any trained individual.

#### 2.3.2 Developmental Coordination Disorder Questionnaire (DCDQ)

The DCDQ (Wilson and Crawford, 2007) is a parent-completed questionnaire that is used to identify motor impairments in children 5 to 15 years old. Parents compare their child’s abilities in 15 activities relative to their TD peers in three different categories: (1) control during movement; (2) fine motor/handwriting; and (3) general coordination. A higher score indicates better motor performance on a scale of 15 to 75. In this study, age-specific cut-off scores were used as specified in the DCDQ manual. The DCDQ has high internal consistency (α = 0.94) and adequate sensitivity (85%) (Wilson et al., 2000; Cairney et al., 2008; Wilson et al., 2009). The DCDQ is the recommended screening tool for DCD according to the international guidelines for identification of children with DCD (Blank et al., 2019).

#### 2.3.3 Conners 3 ADHD Index (Conners 3 AI)

The Conners 3 ADHD Index is parent-completed questionnaire that aids health care professionals in determining whether a child does or does not have ADHD symptoms (Conners, 2009). This norm-referenced assessment is based on a large North American sample. It is one of the most commonly used screening tools to assess ADHD symptoms in both research and clinical settings (Conners, 2009). A score over 70 indicates clinically significant attentional difficulties. The Conners 3 ADHD Index has high internal consistency (α = 0.90), high predictive value, and mean test–retest reliability of 0.83 (Morales-Hidalgo et al., 2017). For the purpose of this study, the Conners 3 ADHD Index was used to quantify the degree of attentional difficulties; higher scores indicate poorer attentional performance.

#### 2.3.4 Sociodemographic questionnaire

A socio-demographic questionnaire was used to collect information regarding participant demographics such as age, sex, history of therapy interventions, medications, and additional diagnoses.

### 2.4 Neuroimaging measures

#### 2.4.1 MRI data acquisition

All brain images were acquired at the Magnetic Resonance Imaging (MRI) Research Facility at BC Children’s Hospital Research Institute in Vancouver, Canada. All children participated in an MRI safety screening and an MRI simulator session to familiarize themselves with the scanning environment (noise, confined space, and head coil). They were also provided with strategies from the research team to help reduce potential anxiety. High resolution isotropic structural scans were obtained on a 3-Tesla General-Electric Discovery MR750 MRI scanner. A T1-weighted 3D structural scan was acquired with the following parameters: three-dimensional spoiled gradient recalled acquisition in steady state (3D SPGR), echo time = 30 ms, repetition time = 3,000 ms, FOV = 256, matrix size = 256 × 256, flip angel = 12^°^, number of slices = 256, slice thickness = 1 mm, interleaved with no gaps (voxel size 0.9375 × 0.9375 × 1 mm). T1-weighted scans were ascertained to permit reliable segmentation of tissues (grey matter, white matter, and cerebrospinal fluid) and reliable identification of underlying regions (Lerch et al., 2017).

#### 2.4.2 Image quality control

All scans were visually inspected for truncation, motion, aliasing-related and other artifacts by trained raters (Krupa and Bekiesińska-Figatowska, 2015; Reuter et al., 2015). Specifically, image quality was assessed for head coverage, wrapping artifact, radiofrequency noise, signal inhomogeneity, susceptibility artifact, and ringing artifact (Reuter et al., 2015). An ordinal score was given to each image based on motion artifacts and image quality (pass, questionable, or fail) using standardized methodology (Harvard Center for Brain Science, 2014). Two trainees assessed the scans independently; the level of agreement for the categorization of each scan assessed by each trainee was 96%. Only scans that passed the final quality check from both trainees were included in the analysis.

Additionally, quantitative measures of motion were calculated using the software package MRIQC (Esteban et al., 2017). In particular, we measured coefficient of joint variation (CJV), where higher values are related to the presence of heavy head motion and large intensity non-uniformity (Ganzetti et al., 2016).

#### 2.4.3 Voxel-based morphometry

##### 2.4.3.1 Image pre-processing

Data were converted from DICOM (Digital Imaging and Communications in Medicine) to NIfTI (Neuroimaging Informatics Technology Initiative) using the dcm2nii tool from MRIcron.^1^ T1 images were processed using voxel-based morphometry (VBM), a computational technique that measures differences in grey matter volume through a voxel-wise comparison (Ashburner and Friston, 2000; Whitwell, 2009). All pre-processing and VBM data analysis were carried out using the Computational Anatomy Tool Box (CAT12, v1742, The Structural Brain Mapping Group, Jena, Germany, http://dbm.neuro.uni-jena.de/cat12/), through Statistical Parametric Mapping 12 software (SPM12, v7771, The Wellcome Centre for Human Neuroimaging, London, United Kingdom, https://www.fil.ion.ucl.ac.uk/spm/) in MATLAB R2020b (Mathworks, Natick, Massachusetts, United States). For image pre-processing, all T1 images were manually registered to the anterior commissure at the origin of the Montreal Neurological Institute (MNI) coordinate system (Jahn, 2019). The co-registered images were then segmented into grey matter (GM), white matter (WM), and cerebrospinal fluid (CSF). As the images were from a pediatric sample, the tissue probability maps of GM, WM, and CSF were obtained using the Template-O-Matic Toolbox (TOM8, http://dbm.neuro.uni-jena.de/software/tom/). All images were included if their weight average Image Quality Rating (IQR) was greater than 80%, corresponding to a “good” image quality. Mean correlations between all volumes were visualized through CAT12. Volumes with a correlation below two standard deviations from the sample mean were again visually inspected for artifacts.

**Figure 2:**
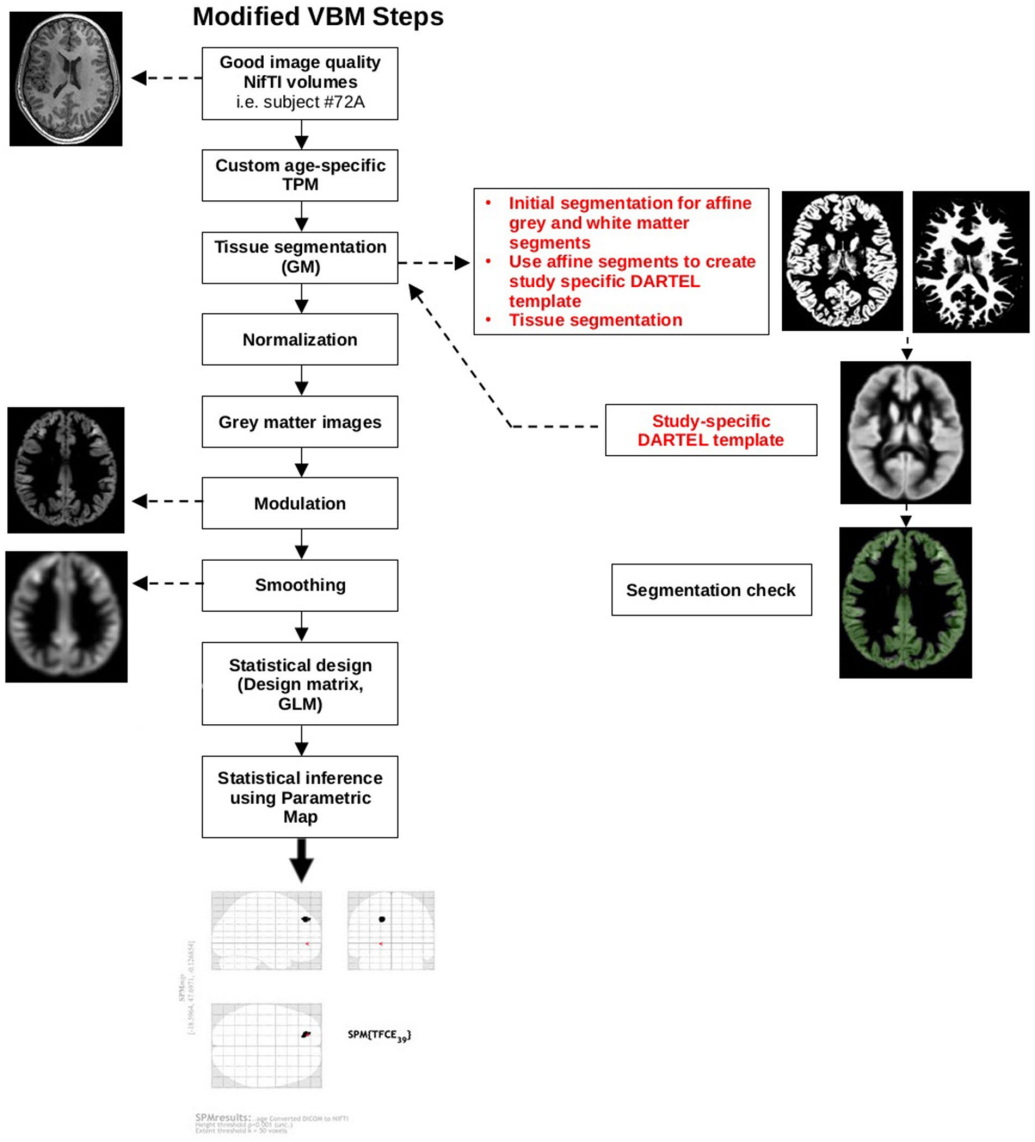
Modified VBM steps according to pediatric sample recommendations. The red text highlights additional steps that were taken to accommodate a pediatric sample. DARTEL, Diffeomorphic Anatomical Registration Through Exponentiated Lie Algebra; GM, grey matter; GLM, general linear model; NIfTI, Neuroimaging Informatics Technology Initiative; SPM, Statistical Parametric Map; TPM, tissue probability map.

Next, good quality affine-registered white and grey matter tissue segments were extracted to construct a customized Diffeomorphic Anatomical Registration Through Exponentiated Lie Algebra (DARTEL) study-specific template registered to the MNI-International Consortium for Brain Mapping (ICBM) space. This alternative to the adult-based template provided by CAT12 was used to achieve a more accurate inter-participant registration to improve the realignment of small inner structures for an overall better segmentation (Good et al., 2001; Yassa and Stark, 2009). This additional step was based on pediatric VBM studies done in other neurodevelopmental disorders that created a study-specific average template for their sample (Reynolds et al., 2017; Wang et al., 2017; Sáenz et al., 2020). Individual images were corrected for bias-field inhomogeneities and segmented into GM, WM, and CSF. The images were then normalized using affine spatial normalization and a further modulation was applied to convert the voxel values of tissue concentration (density) to measures of volume. Finally, the normalized GM maps were smoothed with an isotropic Gaussian kernel (full width at half maximum = 6 mm). Total intracranial volume (TIV) was calculated from the GM, WM, and CSF images for each participant using CAT12 module “Total intracranial volume.” Figure 2 provides a schematic of the modified VBM pipeline.

##### 2.4.3.2 Computational anatomy toolbox (CAT12)

The Structural Brain Mapping Group at the University of Jena (Jena, Germany) designed the automatic and easy-to-use toolbox CAT12 as an extension to the SPM software. CAT12 follows a standard VBM analysis pipeline similar to VBM8. Since our sample’s IQR ranged from 80 to 90%, we used segmentation through SPM’s extension CAT12 rather than FreeSurfer or FSL as SPM produces a more robust segmentation for those with limited image quality (Fellhauer et al., 2015). When compared to previous toolboxes, CAT12 provided a more accurate and robust volumetric analysis (Farokhian et al., 2017) and advanced segmentation tool (Tavares et al., 2020). It has also been used in neurodevelopmental disorders that commonly co-occur with DCD (Wang et al., 2017; Mei et al., 2020; Sáenz et al., 2020) where the workflow was adapted to accommodate a pediatric population as recommended for VBM analysis.

### 2.5 Statistical analysis

#### 2.5.1 Participant characteristics

Participant characteristics were analysed using Jeffreys’s Amazing Statistics Program (JASP https://jasp-stats.org/). The Chi-squared test was used to compare sex distribution between groups. To compare group differences in age, TIV, MABC-2 (motor measure), Conners 3 ADHD Index (attentional difficulties measure), and DCDQ, we used two-tailed Mann–Whitney-U test since Levene’s test and Shapiro–Wilk indicated violation of assumptions of equal variance (*p* < 0.001) and normality (*p* < 0.001), respectively.

#### 2.5.2 VBM statistical analysis

All statistical models were designed with general linear modeling through SPM. Individual participant smoothed grey matter volumes were entered into a second level analysis to estimate differences between DCD vs. TD group using a one-way Analysis of Variance (ANOVA) design. TIV (centered to overall mean) was used as a covariate/nuisance variable as recommended in VBM analysis to account for inter-participant differences. While a two-sample *t*-test was inputted into the statistical design, the output was an ANCOVA, with TIV entered as a covariate. No significant differences between age (*p* = 0.40) or sex (*p* = 0.15) were observed between groups. Subsequently, these variables were not included as covariates in the analysis to conserve degrees of freedom. Threshold-Free Cluster Enhancement (TFCE) thresholding was conducted using the TFCE Toolbox Version r214^2^ with 5,000 permutations (Smith method) with unequal variance (DCD vs. controls) with an E = 0.5 and H = 0.2. Structural images were analyzed using TFCE due to its increased sensitivity compared to voxel-or cluster-based statistics (Smith et al., 2009; Salimi-Khorshidi et al., 2011; Radua et al., 2014). Statistical significance was assessed with the permutation test included in SPM.

Initially, we had planned a regression analysis to examine if MABC-2 and Conners 3 ADHD Index scores predicted grey matter volume; however, MABC-2 and Conners 3 ADHD Index scores were moderately negatively correlated (*r* = −0.66, *p* < 0.001). Instead, two independent regression analysis were used to examine the relationship between grey matter volume and clinical measures of motor function (MABC-2) and attention difficulties (Conners 3 ADHD Index), respectively, while controlling for the effect of intracranial volume.

TFCE and an alpha level of 0.05 were used to help account for type 1 errors. All results are reported with TFCE thresholding; however, they are uncorrected for multiple comparisons (no p_FDR-corrected_ or p_FWE-corrected_) due to the small sample size. Results are presented at *p* < 0.001 with cluster size threshold at 50 voxels. Cluster size threshold was based on current literature regarding cluster thresholding. Given our *N* < 50, we opted for a more stringent cluster threshold of 50 compared to lower thresholds of 10 (Lieberman and Cunningham, 2009; Woo et al., 2014). This is also comparable to previous publications of cerebellar VBM with samples of children with neurodevelopmental disabilities (D’Mello et al., 2015).

## 3 Results

### 3.1 Final sample

**Figure 3:**
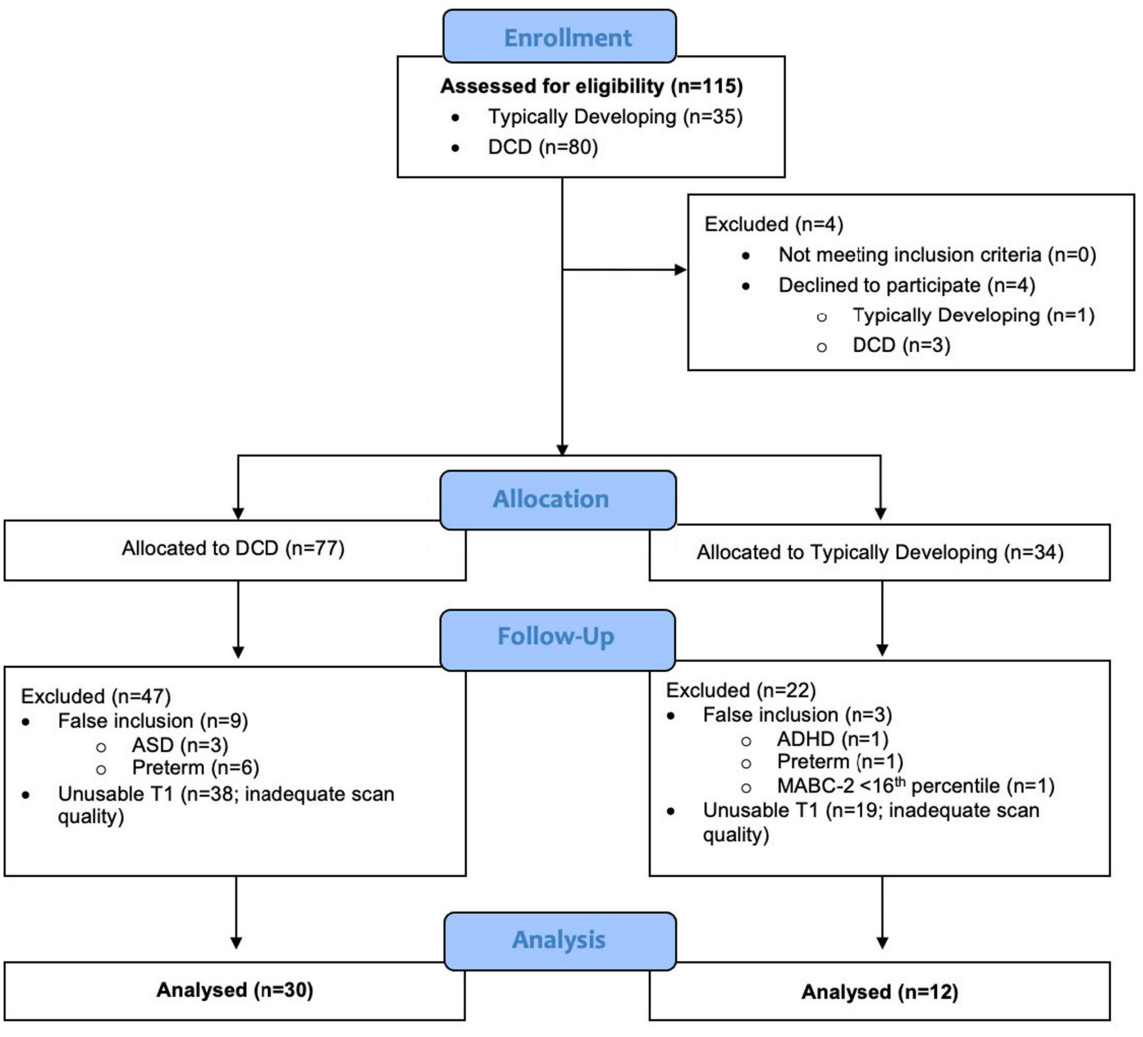
Flow diagram of participant inclusion and exclusion for voxel-based morphometry analysis.

This study recruited 115 children (TD = 35; DCD = 80), from whom 73 were excluded because they either declined to participate (*n* = 4), were later determined to have exclusionary diagnoses or to have been born preterm (*n* = 11), did not meet inclusion criteria (*n* = 1), or had insufficient data quality for VBM analysis (*n* = 57) (Figure 3). Due to the smaller than anticipated sample size, the DCD (*n* = 15) and DCD + ADHD (n = 15) groups were combined; they did not differ significantly in terms of age, sex distribution, MABC-2 subtest and total scores, or Conners 3 ADHD Index scores (all *p* > 0.05). Our final sample included 30 children with DCD [mean (SD) age: 9.9 (1.5) years] and 12 TD children [mean (SD) age: 10.3 (1.5) years]. The majority of participants (74%) were male (Table 1).

**Table 1:** Description of Cohort (*N* = 42).

Clinical characteristics	DCD (*N* = 30) *N* (%) or Mean (SD)	TD (*N* = 12) *N* (%) or Mean (SD)	*p*-value
Male	24 (80)	7 (58)	0.15
Age at MRI (years)	9.9 (1.5)	10.3 (1.5)	0.40
MABC-2 (percentile)	6.1 (7.4)	64.2 (25.5)	<0.001
Conners 3 ADHD Index (t-scores)	84.8 (9.7)	56.5 (11.7)	<0.001
Total intracranial volume (L)	1.53 (0.17)	1.52 (0.08)	0.98

ADHD, attention deficit hyperactivity disorder; DCD, developmental coordination disorder; L, litres; MABC-2, Movement Assessment Battery for Children -2nd edition; MRI, magnetic resonance imaging; SD, standard deviation.

Children (both TD and DCD) whose data were excluded due to motion had an average CJV of 0.73 (± 0.13 SD), while those that were kept had an average CJV of 0.60 (± 0.09 SD). These values were significantly different [*p* < 0.001; 95%CI = (0.09, 0.16)]. Of the participants that were included for analysis, there was no difference in CJV between the TD and DCD cohorts. Furthermore, for the children included for analysis, no correlation was found between CJV and MABC-2 scores [95%CI = (−0.26, 0.06)].

### 3.2 Participant characteristics

Demographic and behavioral characteristics of the sample are shown in Table 1. As expected, the mean total MABC-2 score was significantly lower in children with DCD compared to the typically developing group, indicating significant motor impairments. In addition, the DCD group had significant attentional difficulties (poorer attentional performance) as indicated by a mean score over 70 on the Conners 3 ADHD Index. This finding is consistent with the literature which suggests children with DCD have significant attentional difficulties and high rates of ADHD (Dewey et al., 2002; Kadesjö and Gillberg, 2008; Goulardins et al., 2015; Lange, 2018). Lastly, our DCD sample included 24 males (80%), which aligns with DCD having a higher prevalence in males compared to females (American Psychiatric Association, 2013).

### 3.3 Grey matter differences between TD vs. DCD

**Figure 4:**
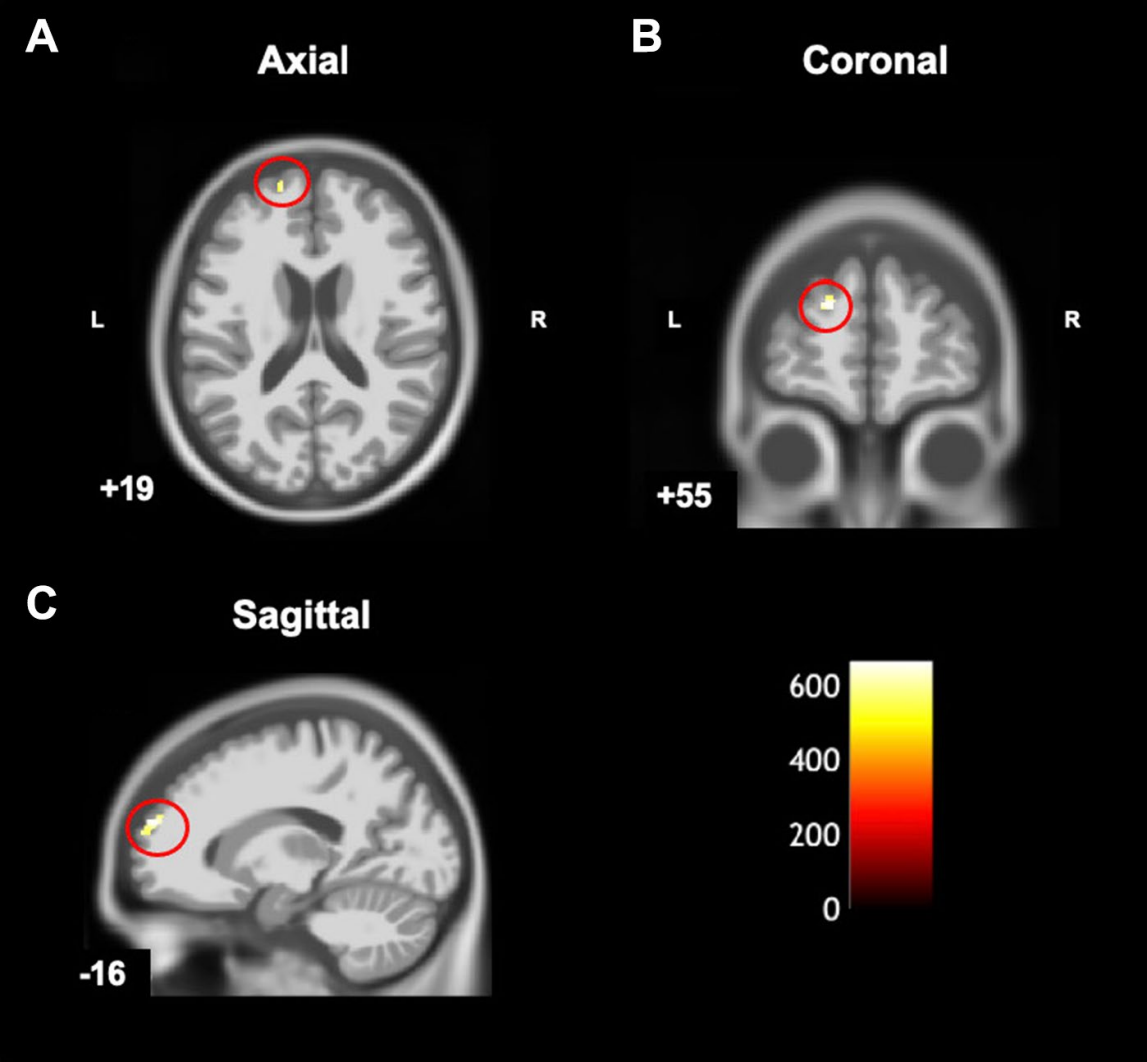
Statistical parametric map superimposed on CAT T1 IXI555 template shows between-group differences with significantly greater grey matter volume (yellow-white region) in children with developmental coordination disorder in comparison to typically developing children (*p* < 0.001 uncorrected). Left superior frontal gyrus in **(A)** Axial; **(B)** Coronal; and **(C)** Sagittal view. Color bar shows t-values post threshold-free cluster enhancement analysis.

Compared to typically developing children, children with DCD had significantly greater grey matter [cluster size (k) >50, p_uncorrected_ ≤ 0.001] in the left superior frontal gyrus (Table 2; Figure 4). There were no regions where children with DCD had lower grey matter volume compared to typically developing children [cluster size (k) <50].

**Table 2:** MNI coordinates for significantly greater grey matter volume in children with developmental coordination disorder compared to typically developing children.

Location	X	Y	Z	TFCE	p_uncorrected_	Cluster size
Left superior frontal gyrus	−16	55	22	666.1	0.001	51
	−16	63	16	599.8	0.001	

### 3.4 Grey matter correlates: motor function and attentional performance

**Figure 5:**
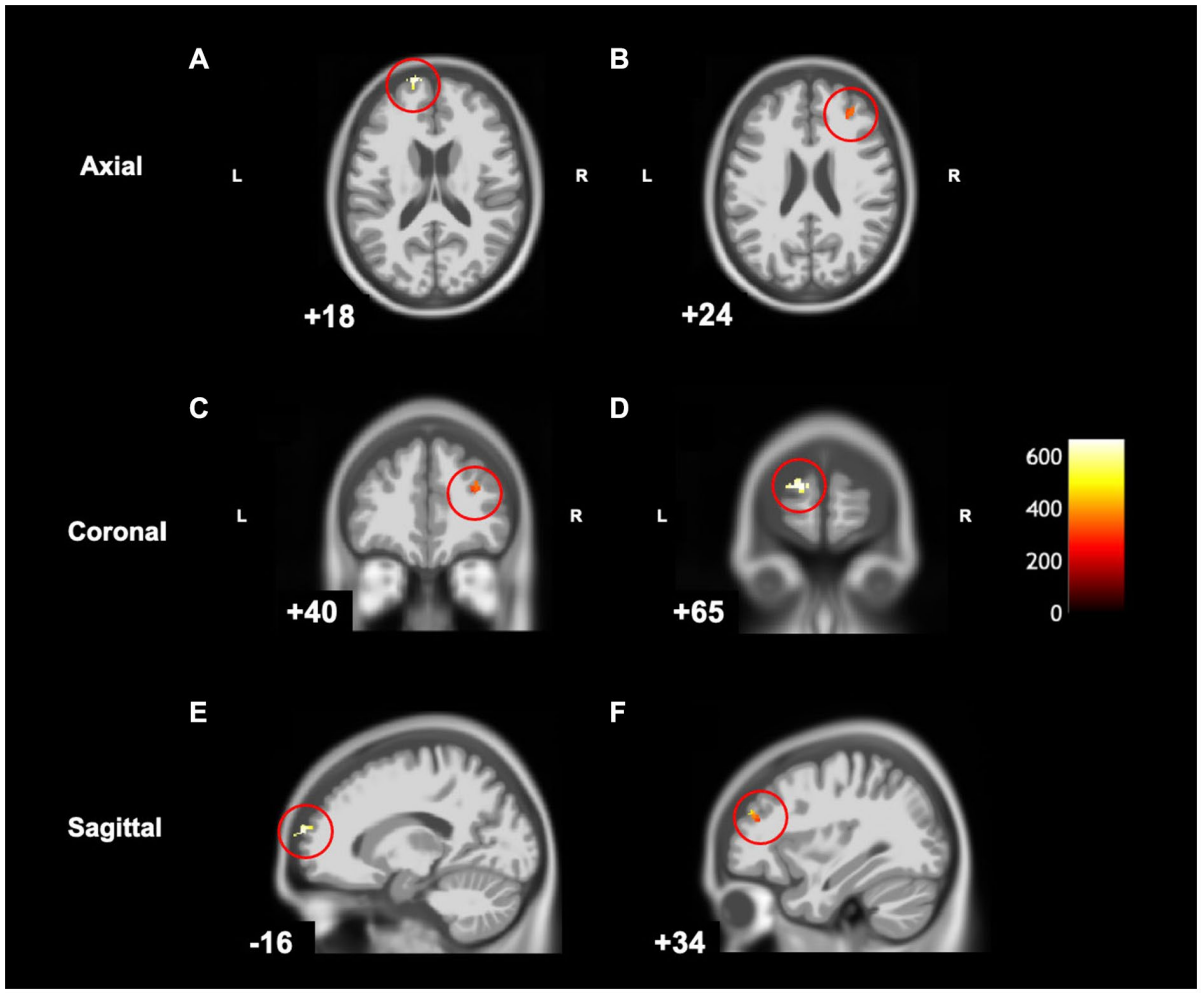
Statistical parametric map superimposed on CAT T1 IXI555 template shows significant negative correlations (yellow and orange) between grey matter and MABC-2 total percentile scores (*p* < 0.001 uncorrected). **(A)** Left superior frontal gyrus and frontal pole; **(B)** Right middle frontal gyrus; **(C)** Right middle frontal gyrus; **(D)** Left frontal pole; **(E)** Left superior frontal gyrus and frontal pole; **(F)** Right middle frontal gyrus. Color bar shows t-values post threshold-free cluster enhancement analysis.

MABC-2 scores were negatively correlated [cluster size (k) >50, p_uncorrected_ ≤ 0.001] with grey matter volume in the left superior frontal gyrus, left frontal pole, and right middle frontal gyrus (Table 3 and Figure 5). Lower MABC-2 scores were related to greater grey matter volume. There were no regions where children with DCD had greater grey matter volume with higher MABC-2 scores [cluster size (k) <50]. The additional clusters (left frontal pole and right middle frontal gyrus) did not overlap with the DCD > TD contrast mentioned above.

**Table 3:** MNI coordinates for correlations between grey matter volumes and MABC-2 percentile scores.

Location	X	Y	Z	TFCE	p_uncorrected_	Cluster size
Left superior frontal gyrus	−14	64	16	665.63	0.001	85
Left frontal pole	−22	69	16	654.00	<0.001	
Right middle frontal gyrus	35	46	28	504.03	0.001	81
	31	39	24	358.29	<0.001	

**Figure 6:**
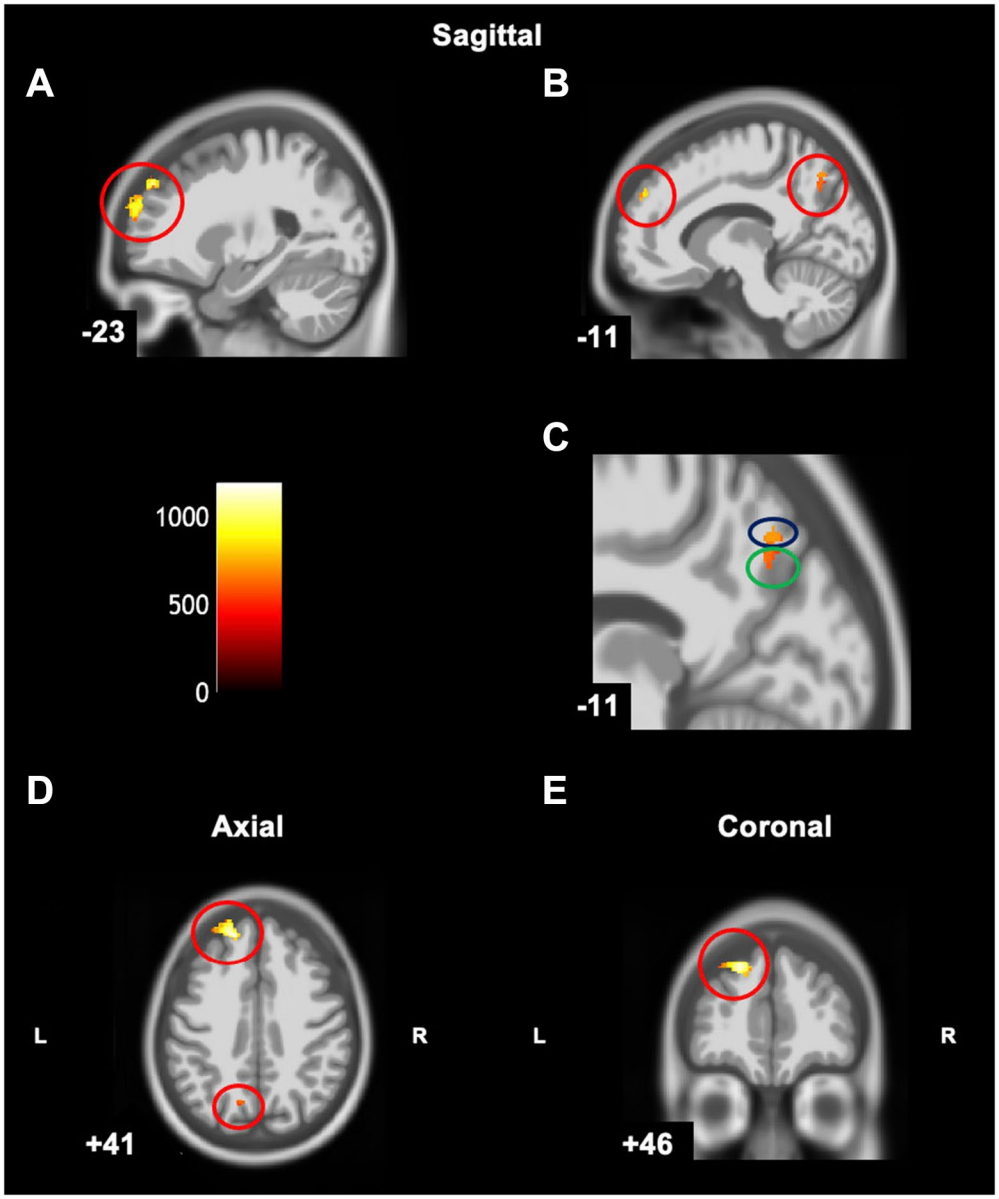
Statistical parametric map superimposed on CAT T1 IXI555 template shows significant positive correlations (yellow and orange) between grey matter and Conners ADHD Index T-Score (*p* < 0.001 uncorrected). **(A)** Left superior frontal gyrus; **(B)** Left superior frontal gyrus, superior parietal lobe, and precuneus; **(C)** Zoomed image of B: Left superior parietal lobe (blue) and left precuneus (green); **(D)** Left superior frontal gyrus; **(E)** Left superior frontal gyrus. Color bar shows *t*-values post threshold-free cluster enhancement analysis.

The Conners 3 ADHD Index T-score was positively correlated with grey matter volume in the left superior frontal gyrus (cluster size (k) >50, p_uncorrected_ < 0.001) (Table 4 and Figure 6), indicating that higher Conners 3 ADHD Index T-scores (greater attentional difficulties/poorer attentional performance) were related to greater grey matter volume. There were no regions where children with DCD had lower grey matter volume with higher Conners ADHD index T-score [cluster size (k) <50]. The additional clusters (left superior parietal lobe and left precuneus) did not overlap with the DCD > TD contrast.

**Table 4:** MNI coordinates for correlations between grey matter volume and Conners 3 ADHD Index T-Score.

Location	X	Y	Z	TFCE	p_uncorrected_	Cluster size
Left superior frontal gyrus	−16	43	37	1192.72	<0.001	663
	−25	60	24	1018.14	<0.001	
	−29	51	37	935.97	<0.001	
Left superior parietal lobe and left precuneus	−13	−65	46	721.47	<0.001	61

## 4 Discussion

This study examined grey matter differences in children with DCD compared to typically developing children. Contrary to our hypothesis, we found that children with DCD had greater grey matter volume compared to TD children. This difference was only found in the left superior frontal gyrus. This result may be clinically significant, as lower MABC-2 scores were significantly correlated with greater grey matter volume in this region, and the same relationship was identified in left frontal pole and right middle frontal gyrus. Greater grey matter volume was also significantly correlated with higher Conners 3 ADHD Index in several regions of the left hemisphere: superior frontal gyrus, superior parietal lobe, and precuneus. These results indicate that greater grey matter volume in these regions is associated with poorer motor skills and worse attentional problems. Our findings do not align with previous structural MRI studies in DCD. Langevin et al. (2014) reported thinner cortex in the right temporal pole and Reynolds et al. (2017) identified smaller grey matter volume in the right frontal lobe, specifically the middle, medial, and superior frontal gyri in children with DCD. These disparate findings may be due to methodological differences between studies. For example, we used a robust VBM analysis (CAT12) and modified pipeline to accommodate a pediatric sample (e.g., Wang et al., 2017). Likewise, Langevin et al. (2014) included participants aged 8 to 17 years, which was a broader age range compared to this study and may have different results due to more variance from brain development across such a broad age range. To discuss our results, we will first highlight typical brain development and then interpret our findings in that context.

Cortical thickness, surface area, and volume are all generally shown to increase from early infancy (Gilmore et al., 2011; Lyall et al., 2014) until late childhood/early adolescence (Giedd et al., 1999; Lenroot et al., 2007; Wierenga et al., 2014; Tamnes et al., 2017). Following this developmental period, normal brain development is characterized by reductions in cortical thickness, grey matter, and surface area, with further thinning and decreases throughout adolescence (Tamnes et al., 2009; Gogtay and Thompson, 2010; Walhovd et al., 2016; Tamnes et al., 2017). Cortical thinning, which has region-specific trajectories, is a hallmark of brain development and evolution (Sowell et al., 2007; Amlien et al., 2014; Tamnes et al., 2017). It is defined as “the decline in thickness of outer layers of the brain that are most evolutionarily advanced in humans and are thought to play particularly important roles in higher levels of information processing and orchestrating actions” (Spear, 2013, p. 3). Tamnes et al. (2017) suggest that cortical thinning is the primary contributor to cortical volume reductions, as surface area exhibits relatively smaller decreases with age. Synaptic pruning and myelination are considered to be two contributors to the complex process of cortical thinning of grey matter that occurs in healthy brain development (Tau and Peterson, 2009; Spear, 2013).

Knowledge about typical brain development is needed to understand brain development in neurodevelopmental disorders, which are characterized by impaired growth, development, or function of the central nervous system (American Psychiatric Association, 2013). Abnormalities in cortical volume and thickness have been reported in a number of neurodevelopmental disorders, including ADHD and ASD (Castellanos et al., 2002; Makris et al., 2006; Nakao et al., 2011; Ha et al., 2015; Lange et al., 2015; Khundrakpam et al., 2017; Liu et al., 2017; Boedhoe et al., 2020; Sáenz et al., 2020). A delay or dysfunction in cortical thinning might explain the anomalies in surface area, volume, and thickness seen in these other disorders (Shaw et al., 2007, 2011; Khundrakpam et al., 2017). Here, we observed higher regional brain volumes in DCD, which we interpret along these lines, and as a dysfunction in cortical thinning. We would also posit that when combined with previous findings, synaptic pruning is the more likely underlying factor in this population. Recent diffusion tensor imaging studies reported white matter differences in DCD relative to typically developing peers (Brown-Lum et al., 2020). The authors found no accompanying differences in radial diffusivity, leading them to conclude that the differences in DCD were unlikely to be related to disrupted myelination (Brown-Lum et al., 2020). Kilroy et al. (2022) also reported no differences in radial diffusivity in DCD compared to TD children. Since typical brain development is associated with increased myelination and synaptic pruning, the combined evidence from the current study (i.e., greater cortical volume in a specific region) and diffusion tensor imaging studies (i.e., unlikely disruption in myelination) (Brown-Lum et al., 2020; Kilroy et al., 2022) suggest that the delay in cortical thinning in children with DCD is likely due to dysfunction or delay in mechanisms responsible for synaptic pruning. Synaptic pruning, which happens between early childhood to adulthood, is defined as the targeted elimination of less functional or extra synapses to improve connections in the brain and is necessary for normal brain development (Lichtman and Colman, 2000; Tessier and Broadie, 2009; Paolicelli et al., 2011; Navlakha et al., 2015; Sakai, 2020). The more a particular synapse is used, the stronger it becomes, which decreases the likelihood of it being eliminated; weaker connections are more susceptible to synaptic pruning (Lichtman and Colman, 2000; Tessier and Broadie, 2009; Paolicelli et al., 2011; Navlakha et al., 2015; Sakai, 2020).

Greater grey matter volume was located in the frontal lobe in children with DCD, specifically in the left superior frontal gyrus. The left superior frontal gyrus is involved in activities that support higher cognitive functions, such as the processing of sensory and motor information (Exner et al., 2002), executive function (e.g., working memory, planning) (Hoffmann, 2013), and spatial cognition (Hopfinger et al., 2000; du Boisgueheneuc et al., 2006; Harms et al., 2013). These functions are consistent with the difficulties reported in children with DCD (Wilson and McKenzie, 1998; Alloway and Temple, 2007; Leonard et al., 2015; Fong et al., 2016; Wilson et al., 2020). Though we were somewhat surprised by the focal nature of this finding, the known functions of the region relate strongly to the clinical picture in DCD. Greater grey matter volume in the left superior frontal gyrus could reflect altered brain development in this specific region, perhaps due to a delay or disruption of synaptic pruning as discussed above; however, future studies are required to confirm this hypothesis.

In addition to the left superior frontal gyrus, motor function was correlated with the right middle frontal gyrus and left frontal pole. The right middle frontal gyrus is suggested to play an important role in re-orientating attention to different environmental stimuli (Japee et al., 2015), where attention plays an important role in motor learning (Song, 2019). The frontal pole cortex, also known as Brodmann area 10, is important in monitoring the outcomes of movements/actions (Tsujimoto et al., 2011). These findings are consistent given the attentional (Dewey et al., 2002; Fliers et al., 2010) and motor learning/planning difficulties in children with DCD (Wilson et al., 2012). A delay or dysfunction in cortical thinning (through decreased pruning) may underlie the greater grey matter in these regions associated with lower MABC-2 scores.

The findings regarding attentional scores follow a similar explanation. Worse attentional symptomatology was correlated with greater grey matter volume in the superior frontal gyrus (discussed above), as well as the superior parietal lobe and precuneus. The superior parietal lobe is involved in manipulating information in working memory (Koenigs et al., 2009) and sensorimotor integration (Wolpert et al., 1998). The precuneus is part of the parietal cortex and is involved in a wide variety of cognitive processes, including internally guided attention and shifting attention in motor imagery tasks (Cavanna and Trimble, 2006). The greater grey matter volume in these regions may be a result of decreased synaptic pruning. In addition to structural differences, a recent study by Rinat et al. (2020) reported altered functional connectivity between the sensorimotor network and the posterior cingulate cortex and precuneus in children with DCD, providing further evidence that these regions are implicated in DCD. Greater grey matter volume has also been observed in ASD (Tang et al., 2014), a common co-occurrence with DCD. In addition, the precuneus has been implicated in other neurodevelopmental disorders that have difficulty with attention (Nakao et al., 2011; Sáenz et al., 2020). Animal models suggest that the consequences of excessive synaptic connections due to a failure of synaptic pruning impairs learning new spatial re-orientations (Afroz et al., 2016); these findings suggest that too many brain connections (synapses) may put limitations on learning potential (Eltokhi et al., 2020) which is consistent with the difficulties presented in children with DCD (Alloway and Archibald, 2008; Tsai et al., 2012).

We would also point out that the ADHD-related findings in this DCD group do not align with many studies in “stand-alone” ADHD, which show greater cortical thinning in prefrontal and frontolimbic regions (e.g., Francx et al., 2016). The findings here may thus be specific to the combined circuitry affected in individuals with both ADHD and DCD, and needs replication. This is also interesting from a transdiagnostic research perspective, perhaps illustrating that across some disorders (and possibly even at some ages), the neural correlates associated with some symptom domains may be unique and not transdiagnostic.

There are several limitations in this study. First, our sample size was much smaller than anticipated so we were unable to control for multiple comparisons. After applying exclusion criteria and stringent quality checks of the 111 scans, our final sample was relatively small (N = 42) and unequal (DCD was nearly double the size of the non-DCD cohort). However, only the highest quality scans were included which increases confidence in the findings and generalizability of the results (Sáenz et al., 2020). Further, we had intended to analyze children with DCD and children with DCD + ADHD separately, but due to the smaller than anticipated sample size, we combined the children into one group. However, the majority of children in our sample had clinically significant ADHD symptoms (regardless of diagnosis), which may have minimized the anticipated group differences. We noted that 5/15 (33%) of children with DCD + ADHD were taking stimulant medication which may have confounded the results, particularly if they had been taking the stimulants for long periods of time (Nakao et al., 2011). In addition, there were some limitations regarding volume-based measures. Since grey matter includes surface area and thickness, each of which have their own developmental trajectories, the interpretation of grey matter volume becomes difficult without examining surface area or thickness individually (Frye et al., 2010). Future studies should continue to explore differences in grey matter volume in children with DCD but in a larger sample and over time to examine if maturation differs from typically developing children. In addition, exploring cortical thickness and volume in the same study would provide more insight into the structural morphology associated with DCD. Likewise, results could be stratified by age, sex and/or medication use to provide further insights (Caviness et al., 1996; De Bellis et al., 2001; Spencer et al., 2013). More longitudinal studies from childhood through adolescence evaluating cortical thickness, volume, and surface area in this population are needed to better delineate structural morphology in DCD. Lastly, to further explore mechanisms of synaptic pruning, animal and molecular studies should be conducted to examine the underlying behavioral and neurological consequences of altered synaptic pruning in this population.

In conclusion, we found that children with DCD had greater grey matter volume in the left superior frontal gyrus, and that greater grey matter volume in this region and other frontal and parietal regions was associated with poorer motor and attentional skills. These findings support the conceptualization of DCD as a neurodevelopmental disorder, as in general, cortical thinning is associated with healthy development and advances in skills and aptitudes. We hypothesize that the greater grey matter volume in superior frontal gyrus may reflect a delay or absence of healthy cortical thinning in DCD, potentially due to altered synaptic pruning as seen in other neurodevelopmental disorders. This study adds to growing evidence that DCD may be related to altered brain development. Additional new research will be needed to determine what factors influence brain development in children with DCD, and which risk factors may be modifiable to potentially prevent this common motor disorder.

## Data availability statement

The raw data supporting the conclusions of this article will be made available by the authors, without undue reservation.

## Ethics statement

The studies involving humans were approved by Children’s and Women’s Health Centre/University of British Columbia Research Ethics Board. The studies were conducted in accordance with the local legislation and institutional requirements. Written informed consent for participation in this study was provided by the participants’ legal guardians/next of kin and participating children assented to take part in this study.

## Author contributions

MM: Formal analysis, Visualization, Data curation, Validation, Writing – original draft. AW: Formal analysis, Visualization, Writing – review & editing. DL: Formal analysis, Writing – review & editing. TV: Formal analysis, Writing – review & editing. JZ: Formal analysis, Visualization, Conceptualization, Funding acquisition, Methodology, Project administration, Resources, Supervision, Writing – review & editing.

## Footnotes

^1^https://www.nitrc.org/projects/mricron

^2^http://dbm.neuro.uni-jena.de/tfce/
